# Identification of a circulating MicroRNA signature to distinguish recurrence in breast cancer patients

**DOI:** 10.18632/oncotarget.10485

**Published:** 2016-07-08

**Authors:** Dezheng Huo, Wendy M. Clayton, Toshio F. Yoshimatsu, Jianjun Chen, Olufunmilayo I. Olopade

**Affiliations:** ^1^ Department of Public Health Sciences, University of Chicago, Chicago, IL, USA; ^2^ Section of Hematology and Oncology, Department of Medicine, University of Chicago, Chicago, IL, USA; ^3^ Department of Cancer Biology, University of Cincinnati, Cincinnati, OH, USA

**Keywords:** MicroRNAs, breast neoplasms, prognosis, serum, real-time polymerase chain reaction

## Abstract

There is an urgent need for novel noninvasive prognostic biomarkers for monitoring the recurrence of breast cancer. The purpose of this study is to identify circulating microRNAs that can predict breast cancer recurrence. We conducted a microRNA profiling experiment in serum samples from 48 breast cancer patients using Exiqon miRCURY microRNA RT-PCR panels. Significantly differentiated miRNAs for recurrence in the discovery profiling were further validated in an independent set of sera from 20 patients with breast cancer recurrences and 22 patients without recurrences. We identified seven miRNAs that were differentially expressed between breast cancer patients with and without recurrences, including four miRNAs upregulated (miR-21-5p, miR-375, miR-205-5p, and miR-194-5p) and three miRNAs downregulated (miR-382-5p, miR-376c-3p, and miR-411-5p) for recurrent patients. Using penalized logistic regression, we built a 7-miRNA signature for breast cancer recurrence, which had an excellent discriminating capacity (concordance index=0.914). This signature was significantly associated with recurrence after adjusting for known prognostic factors, and it was applicable to both hormone-receptor positive (concordance index=0.890) and triple-negative breast cancers (concordance index=0.942). We also found the 7-miRNA signature were reliably measured across different runs of PCR experiments (intra-class correlation coefficient=0.780) and the signature was significantly higher in breast cancer patients with recurrence than healthy controls (p=1.1×10^−5^). In conclusion, circulating miRNAs are promising biomarkers and the signature may be developed into a minimally invasive multi-marker blood test for continuously monitoring the recurrence of breast cancer. It should be further validated for different subtypes of breast cancers in longitudinal studies.

## INTRODUCTION

While nearly 5% of breast cancer patients are diagnosed at stage IV (de novo metastatic breast cancer) in the United States [1], approximately 20-30% of early stage breast cancer cases will eventually experience recurrence and develop distant metastasis [2]. Inability to control disease at sites of metastasis is the cause of all breast cancer related deaths. In the United States, it is estimated that nearly 40,000 women per year or 108 women per day die from breast cancer [3], but there is currently no acceptable method for monitoring patients who are likely to progress. Recent advances in the identification of druggable targets based on molecular pathways, which represent the “Achilles heel” of cancer cells, could provide unique opportunities to treat patients with early recurrence before they become symptomatic [4]. Therefore, there is an urgent need to identify novel biomarkers that can predict which patients will progress, either at diagnosis or before clinical manifestation of recurrence.

MicroRNAs (miRNAs) in circulation have good potential to serve as prognostic and predictive biomarkers for breast cancer. MiRNAs are small, non-coding RNA molecules, ~22 nucleotides in length. They bind to complementary sequences in the 3′UTR of multiple target mRNAs, usually resulting in their silencing, and thus regulating gene expression in a wide range of biological and pathological processes [5]. Dysregulation of miRNA expression has been linked to carcinogenesis [6–8]. Because circulating miRNAs are stable after sample collection and can be uniformly amplified and quantified, they represent a class of emerging biomarkers for breast cancer prognosis [9, 10]. Expression of miRNAs in serum or plasma have been examined in breast cancer, but most previous studies often started with few candidate miRNAs and have generated inconsistent results [11–15]. Two previous studies have investigated whole miRNA profile in circulation using microRNA arrays; one study compared metastatic breast cancer with healthy controls, and found circulating miRNAs can indicate status of circulating tumor cells in patients with metastatic breast cancer; another study identified a miRNA signature for predicting relapse in triple-negative breast cancer patients [16, 17]. In this study, we used a discovery/validation approach and systematically examined human miRNome in serum samples to identify a panel of circulating miRNAs that can differentiate patients with breast cancer recurrences from those without recurrences. We developed a miRNA signature for recurrence and examined its reproducibility.

## RESULTS

### Expression profiling of microrna in serum among patients with or without recurrences

**Figure 1:**
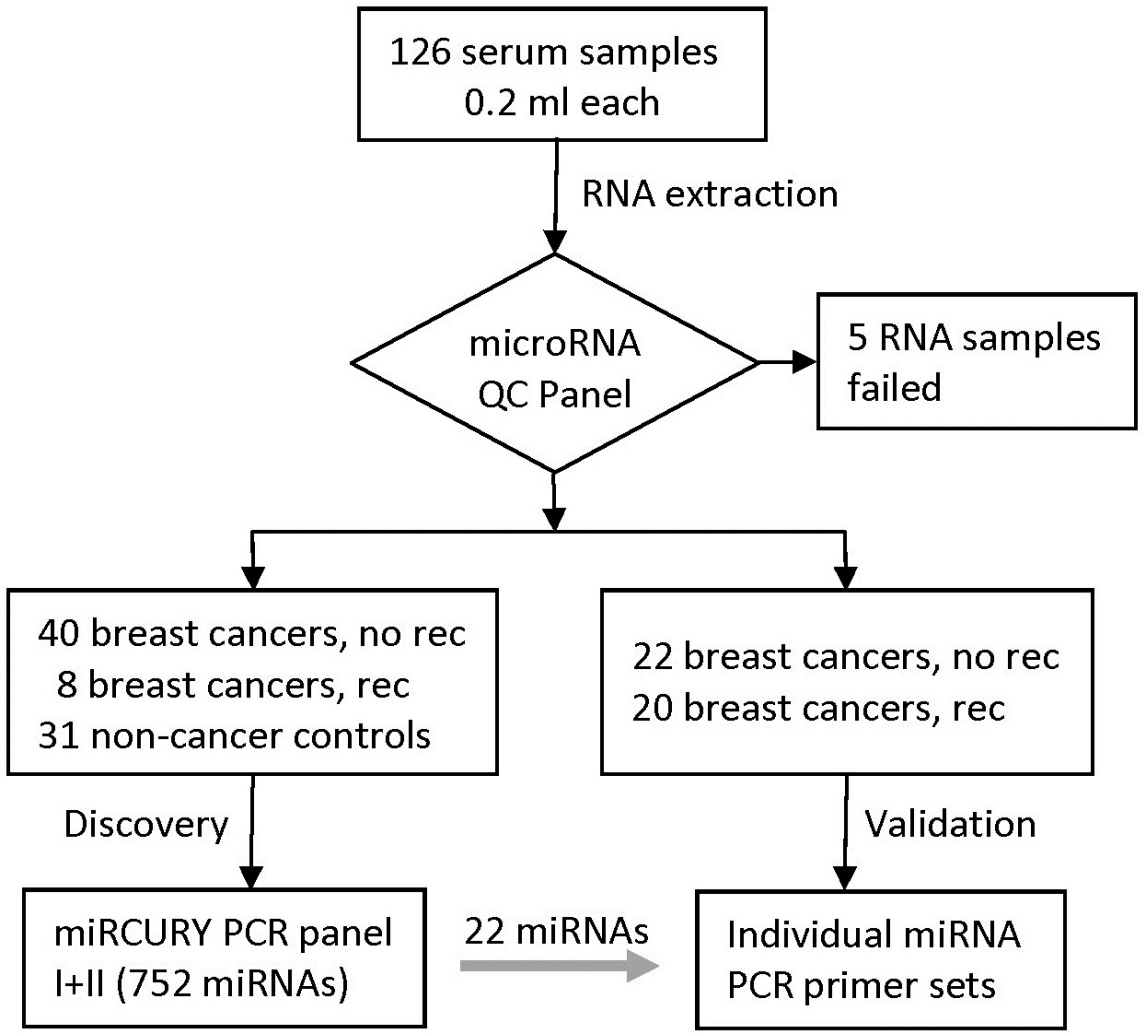
Study design and diagram of sample flow

The study design and sample flow are shown in Figure 1. Serum samples from 126 women were processed to extract RNAs and five samples were excluded because of low RNA quality. Of the remaining samples, 90 women were breast cancer patients and 31 women were non-cancer controls. Of the 90 breast cancer patients, 28 had recurrences, including eight patients with locoregional recurrences and 20 with distant metastases (Table 1). Demographic and clinical factors were similar between the two groups except that the recurrent group had higher grade and higher proportion of HER2+ disease than patients without recurrence. For the 62 patients without recurrence (the “NoRec” group), sera were collected at median of 26 days after diagnosis. They were randomly divided into the discovery phase (40 patients) and validation phase (22 patients). For the 28 patients with recurrence, the median time from diagnosis to recurrence was 2.3 years. Of them, 18 patients contributed sera collected around the time of recurrence (the “Rec-A” group, median = 35 days around recurrence) and were divided into the discovery phase (8 patients) and validation phase (10 patients). There were 10 recurrent patients who contributed serum samples around cancer diagnosis (the “Rec-B” group, median = 70 days after diagnosis) and they were included in the validation phase. There were two *BRCA1* and one *BRCA2* mutation carriers, and none of them had recurrent disease during follow-up.

**Table 1:** Characteristics of breast cancer patients

Characteristic	Levels	Recurrence (n=28)	No recurrence (n=62)	P value*
Age, mean (SD)		47.5 (11.5)	50.4 (11.7)	0.28
Race	White American	11	39	0.057
	African American	16	20	
	Asian American	1	3	
T stage	T1	10	30	0.065
	T2	9	20	
	T3	2	11	
	T4	4	1	
N stage	N0	8	34	0.10
	N1	14	23	
	N2	2	4	
	N3	2	1	
AJCC stage group	I	6	19	0.23
	II	9	29	
	III	9	14	
	IV	1	0	
Histology	Ductal	24	44	0.38
	Lobular	0	5	
	Ductal & lobular	2	6	
	Others	2	6	
Grade	G1	0	5	0.035
	G2	5	25	
	G3	20	31	
Estrogen receptor (ER)	Negative	18	29	0.17
	Positive	10	33	
Progesterone receptor	Negative	20	32	0.11
(PR)	Positive	8	30	
HER2	Negative	21	58	0.031
	Positive	7	4	
Triple negative	No	15	35	0.82
	Yes	13	27	
Mutation status	BRCA1 carrier		2	
	BRCA2 carrier		1	
Type of recurrence	Locoregional	8		
	Distant metastasis	20		
Site of distant metastasis	Bone	8		
	Distant lymph nodes	8		
	Lung	7		
	Brain	3		
	Liver	3		
	Pleura	2		
Phase of the study	Discovery	8	40	
	Validation	20	22	

* t test or Fisher's exact test for continuous and categorical characteristics, respectively

**Figure 2:**
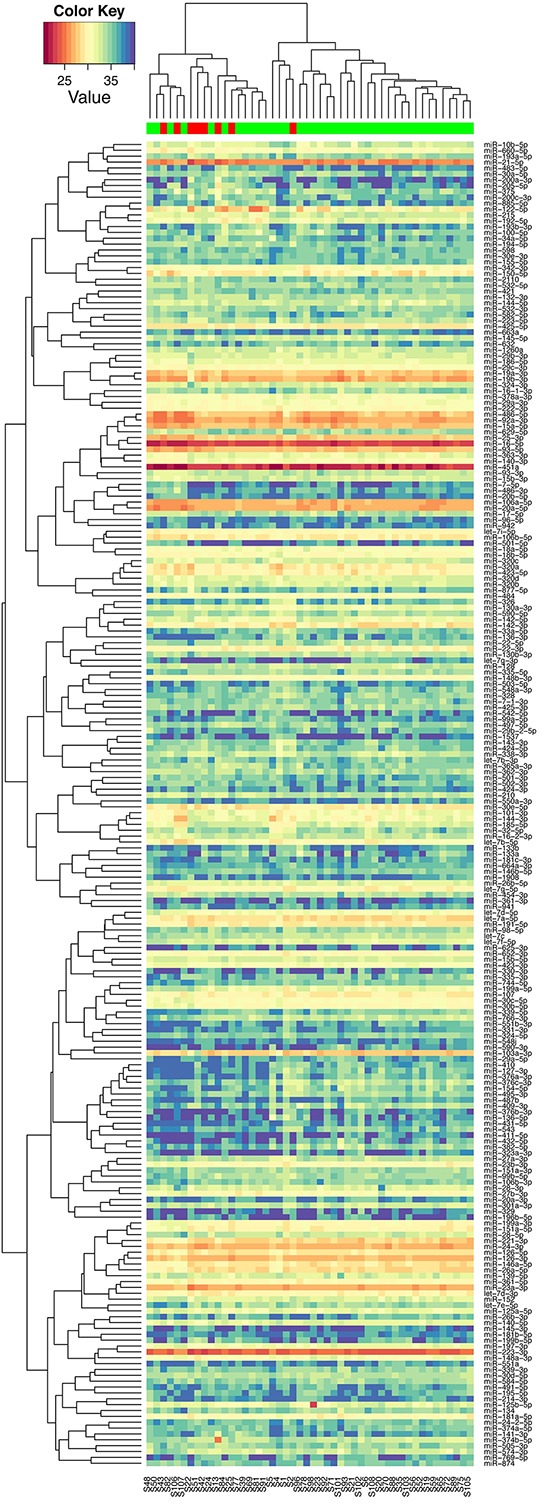
Unsupervised hierarchical clustering with Pearson distance metric, based on 226 detectable miRNAs in serum (red: samples at recurrence from patients with recurrence; green: samples at diagnosis from patients without recurrence)

**Figure 3:**
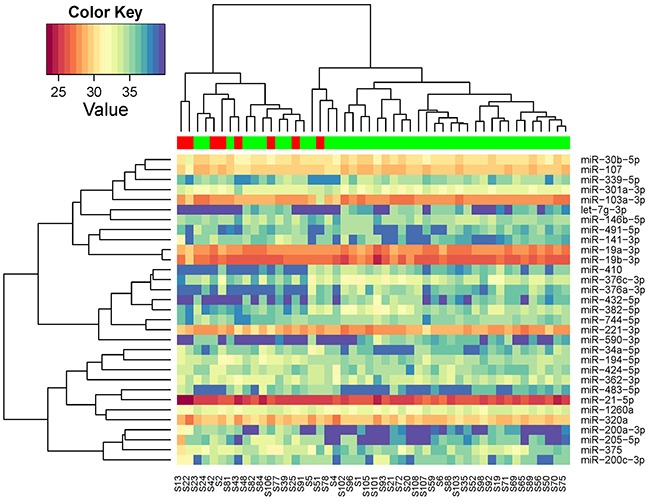
Unsupervised hierarchical clustering with Pearson distance metric, based on the 31 miRNAs that were differentiated expressed between breast cancer patients with and without recurrence in discovery phase (red: samples at recurrence from patients with recurrence; green: samples at diagnosis from patients without recurrence)

Of the 752 miRNAs measured in the discovery phase, 226 could be detected in at least half of the serum samples and thus included in further analysis. Unsupervised clustering analysis showed that these miRNAs self-organized samples into two clusters, with one cluster mainly consisting of patients without recurrences (Figure 2). To identify differentially expressed miRNAs, we conducted moderated t tests and found 31 miRNAs were statistically significantly different between patients with and without recurrences. There was an enrichment of recurrence-associated miRNAs with false discovery rate ranges from 0.35 to 0.0017 for top 31 miRNAs. Again, the miRNA profile of the 31 miRNAs could organize patients into two clusters: one for recurrence and the other for non-recurrence (Figure 3).

As illustrated in Figure 3, some of these 31 miRNAs were correlated with each other. To avoid redundant information, we only chose one miRNA with higher reliability if two were highly correlated for further validation. For example, miR-221-3p and miR-744-5p was correlated with r=0.70 and we chose miR-211-3p as it has lower Cq values. Clustering analysis of the 19 selected miRNAs showed that they can represent the main data structure of the 31 miRNAs (Supplementary Figure 1). We also added one miRNA (miR-411-5p) that was marginally significant in our study but highlighted in a previous study [14]. As a result, a total of 20 miRNAs were selected for testing in the validation phase. In addition, two miRNAs (miR-361-5p and miR-186-5p) were chosen as endogenous controls for qRT-PCR.

### Replication of microRNA signature for breast cancer recurrence

**Figure 4:**
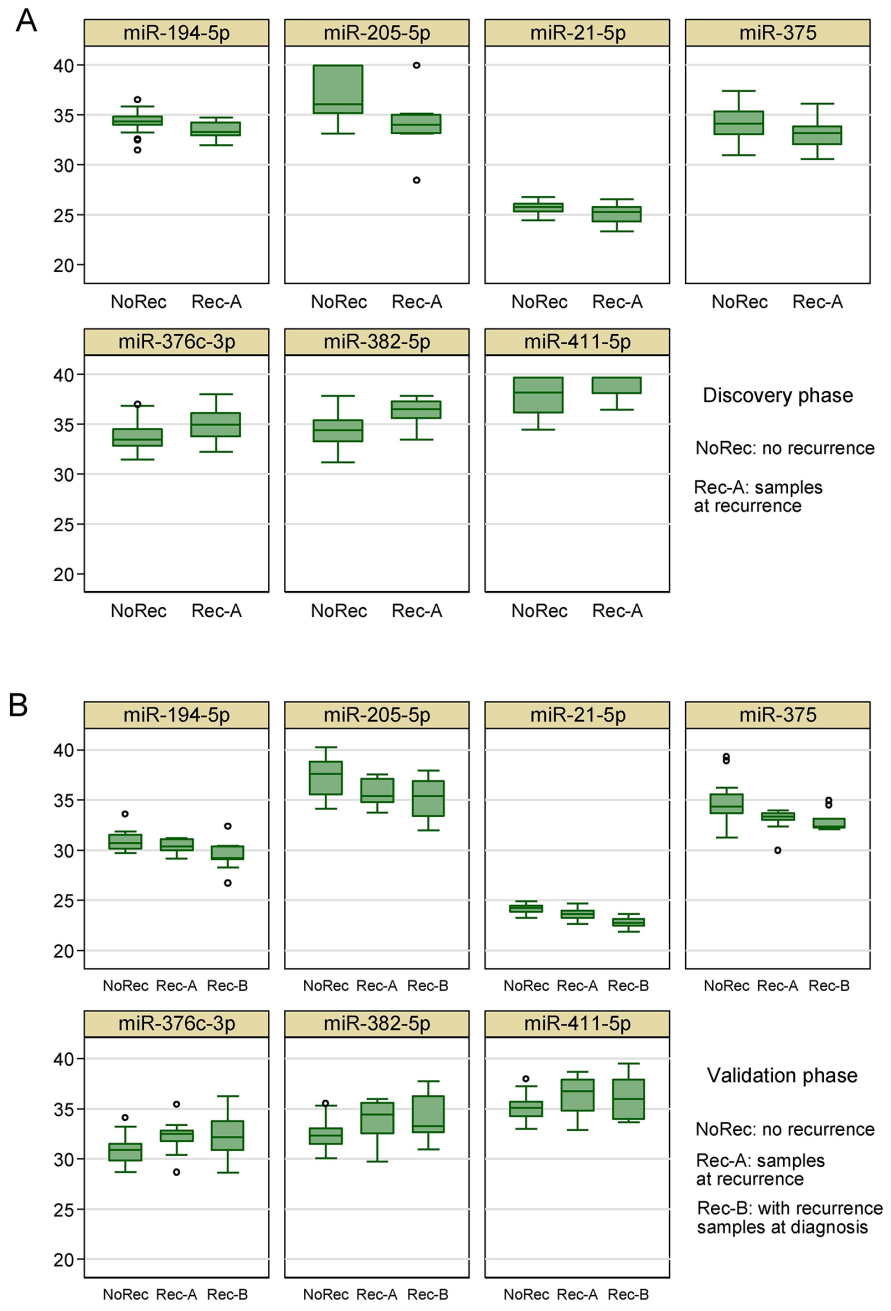
Box plots of the 7 circulating miRNAs associated with tumor recurrence in **A.** the discovery phase and **B.** the validation phase.

In the validation phase, we found that seven out of the 20 miRNAs were significantly associated with recurrence and the direction of association was consistent with that in the discovery phase (Table 2). Figure 4 shows the distribution of these 7 miRNAs in both discovery and validation phase. For four miRNAs (miR-194-5p, miR-205-5p, miR-21-5p, and miR-375), the expressions in samples at recurrence (“Rec-B”) or samples at diagnosis (“Rec-A”) for patients with recurrences were consistently higher than that in patients without recurrence. For three miRNAs (miR-376c-3p, miR-382-5p, and miR-411-5p), the expressions in samples at recurrence or samples at diagnosis for patients with recurrences were consistently lower than that in patients without recurrence. Therefore, we combined samples at diagnosis and recurrence for patients with recurrence together for further analysis. Another five miRNAs (miR-19a-3p, miR-200a-3p, miR-221-3p, miR-103a-3p, and miR-30b-5p) were not statistically significant in the validation phase, but the directions of association were the same as those observed in the discovery phase. Consistent with the discovery phase, the expression of the two endogenous control miRNAs that we chose (miR-361-5p and miR-186-5p) were very similar between patients with and those without recurrence in the validation phase.

**Table 2:** Candidate miRNAs selected in discovery phase and their results in validation phase

	Discovery phase			Validation phase				Pooled analysis	
	Mean Cq	FC*	P value	Mean Cq	FC*	FC†	P value	FC‡	P value
miR-103a-3p	28.0	0.43	0.0011	25.9	0.86	1.03	0.69	0.70	**0.013**
miR-107	29.5	0.68	0.0095	31.1	0.99	1.37	0.45	0.94	0.65
miR-1260a	32.8	1.57	0.021	31.2	0.73	1.47	0.11	1.21	0.20
miR-141-3p	36.3	2.73	0.044	35.3	1.05	1.52	0.58	1.70	0.071
miR-146b-5p	34.8	0.61	0.036	32.6	1.54	1.80	0.025	1.13	0.42
**miR-194-5p**	**34.2**	**1.86**	**0.014**	**30.4**	**1.39**	**2.77**	**0.0025**	**1.92**	**0.00018**
miR-19a-3p	27.7	0.68	0.020	25.1	0.82	0.86	0.47	0.77	**0.013**
miR-200a-3p	**37.4**	**12.47**	**7.5E-06**	36.4	1.22	2.37	0.10	**3.69**	**7.1E-05**
miR-200c-3p	35.4	2.48	0.05	34.5	1.57	0.79	0.18	1.52	0.108
**miR-205-5p**	**36.4**	**7.04**	**0.0036**	**36.3**	**2.81**	**4.23**	**0.0071**	**4.55**	**8.5E-05**
**miR-21-5p**	**25.6**	**1.55**	**0.018**	**23.7**	**1.45**	**2.59**	**5.9E-07**	**1.78**	**5.2E-07**
miR-221-3p	28.5	0.67	0.049	27.1	0.87	0.67	0.19	0.72	**0.014**
miR-301a-3p	32.8	0.68	0.043	30.5	1.01	1.33	0.53	0.94	0.69
miR-30b-5p	30.3	0.67	0.023	28.1	0.81	0.92	0.53	0.78	**0.026**
miR-320a	29.2	1.44	0.05	26.1	0.76	0.84	0.35	1.01	0.96
**miR-375**	**34.1**	**2.21**	**0.05**	**33.9**	**3.15**	**3.40**	**0.0038**	**2.81**	**0.00014**
**miR-376c-3p**	**33.9**	**0.41**	**0.027**	**31.5**	**0.38**	**0.36**	**0.024**	**0.38**	**0.00051**
**miR-382-5p**	**34.7**	**0.27**	**0.0020**	**33.2**	**0.37**	**0.33**	**0.026**	**0.32**	**7.6E-05**
**miR-411-5p**	**38.0**	**0.49**	**0.15**	**35.6**	**0.36**	**0.46**	**0.038**	**0.44**	**0.006**
miR-424-5p	34.7	1.89	0.027	29.4	0.57	0.59	0.015	0.88	0.11

Note: quantitation cycle (Cq); fold change (FC) = 2^ΔCq^ ; significant, consistent validated results are in bold

* FC: fold change comparing samples at recurrence for patients who had recurrent diseases vs. samples at diagnosis for patients without recurrence

† FC: fold change comparing samples at diagnosis for patients who had recurrence vs. samples at diagnosis for patients without recurrence

‡ FC: fold change comparing samples from patients with recurrence vs. patients without recurrence

**Figure 5:**
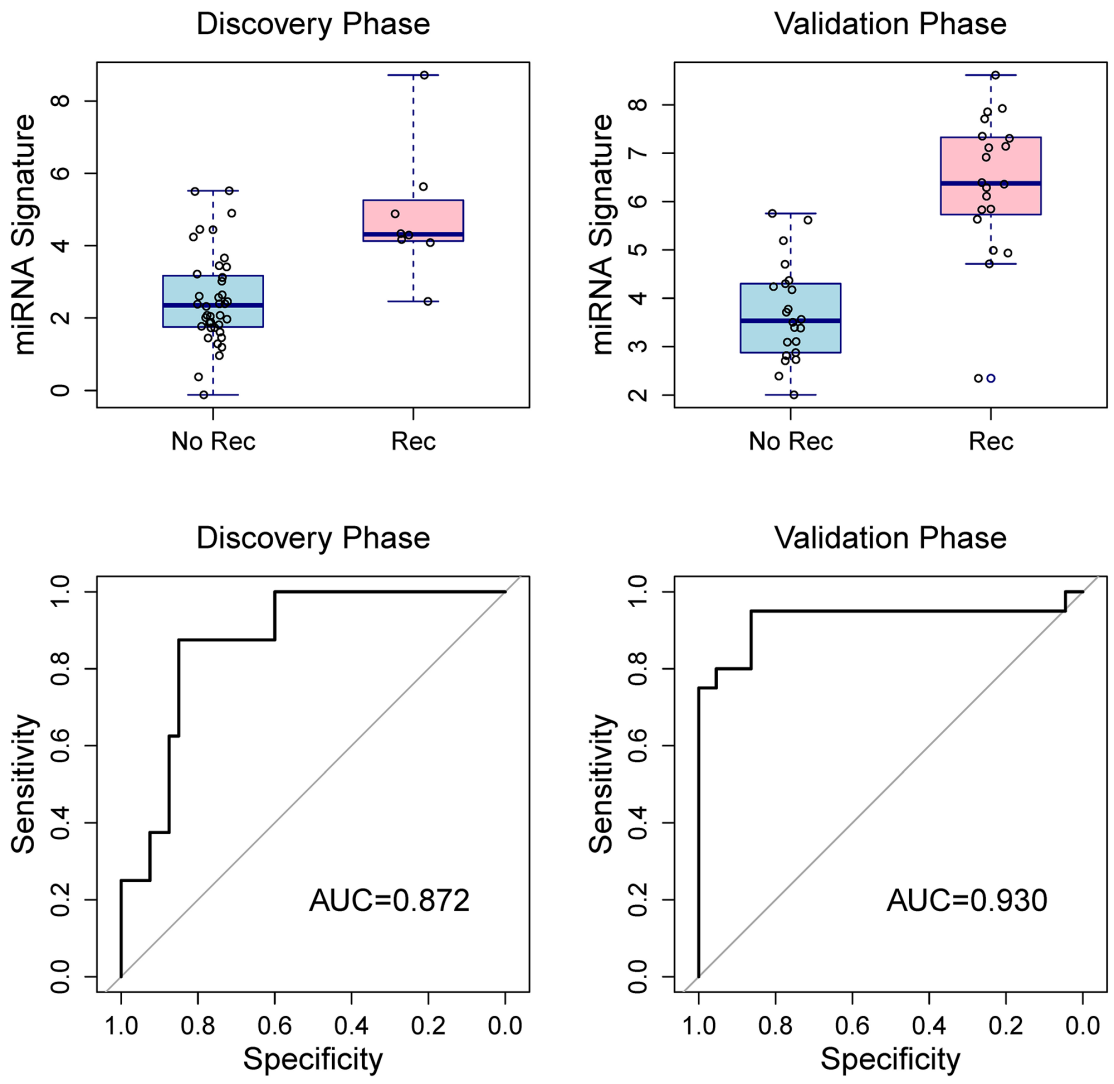
Box plots of the 7-miRNA signature in the discovery and validation phases (top two panels) and receiver operating characteristic curves for penalized logistic regressions (bottom two panels) show the discriminating capacity of the 7-miRNA signature. Rec, recurrence; NoRec, no recurrence

There were weak to moderate correlation among the seven validated miRNAs (Table 3). Using ROC curve, we estimated the discriminating capacity of individual miRNAs and the AUC ranged from 0.65 to 0.86 (Table 4). Using penalized logistic regression, we developed a miRNA signature to discriminate breast cancer patients with recurrences and without (Table 5). After adjusting for age, race, tumor size, lymph node status, histologic grade, and HER2 status, the miRNA signature was still significantly associated with breast cancer recurrences. In addition, excluding the 3 BRCA1/2 mutation carriers did not change the results substantially. The AUC for the 7-miRNA signature was 0.872 in the discovery phase, and 0.930 in the validation phase (Figure 5). The AUC of pooling samples from the two phases was 0.914, suggesting that the 7-miRNA signature has better discriminating capacity than individual miRNAs. Using a signature score of 4.2 as the cutoff point, the sensitivity was 92.9% and the specificity was 77.4%.

**Table 3:** Matrix of correlation coefficients among the 7 validated miRNAs

	miR-194-5p	miR-205-5p	miR-21-5p	miR-375	miR-376c-3p	miR-382-5p
miR-194-5p	1					
miR-205-5p	0.34*	1				
miR-21-5p	0.08	0.29*	1			
miR-375	0.32*	0.50*	0.32*	1		
miR-376c-3p	−0.41*	−0.34*	−0.14	−0.33*	1	
miR-382-5p	−0.49*	−0.28	−0.10	−0.30*	0.72*	1
miR-411-5p	−0.52*	−0.09	−0.00	−0.27	0.51*	0.44*

* p<0.05

**Table 4:** Area under ROC curve for individual miRNAs

	Discovery phase	Validation phase
miR-194-5p	0.763	0.730
miR-205-5	0.813	0.759
miR-21-5p	0.688	0.864
miR-375	0.694	0.814
miR-376c-3p	0.716	0.741
miR-382-5p	0.819	0.743
miR-411-5p	0.653	0.700

**Table 5:** Penalized logistic regression of 7 miRNAs and the distribution of miRNA signature

	Log odds ratio	P value
Penalized logistic regression		
miR-194-5p	0.431	
miR-205-5p	0.261	
miR-21-5p	0.788	
miR-375	0.198	
miR-376c-3p	−0.176	
miR-382-5p	−0.160	
miR-411-5p	−0.154	
miRNA signature (unadjusted)	1.275	2.0E-06
miRNA signature (adjusted*)	1.261	3.6E-05
Distribution of miRNA signature	mean ± SD	
Recurrent group	5.93 ± 1.67	
No recurrent group	2.94 ± 1.31	

* adjusted for age, race, tumor size, lymph node status, histologic grade, and HER2 status

**Figure 6:**
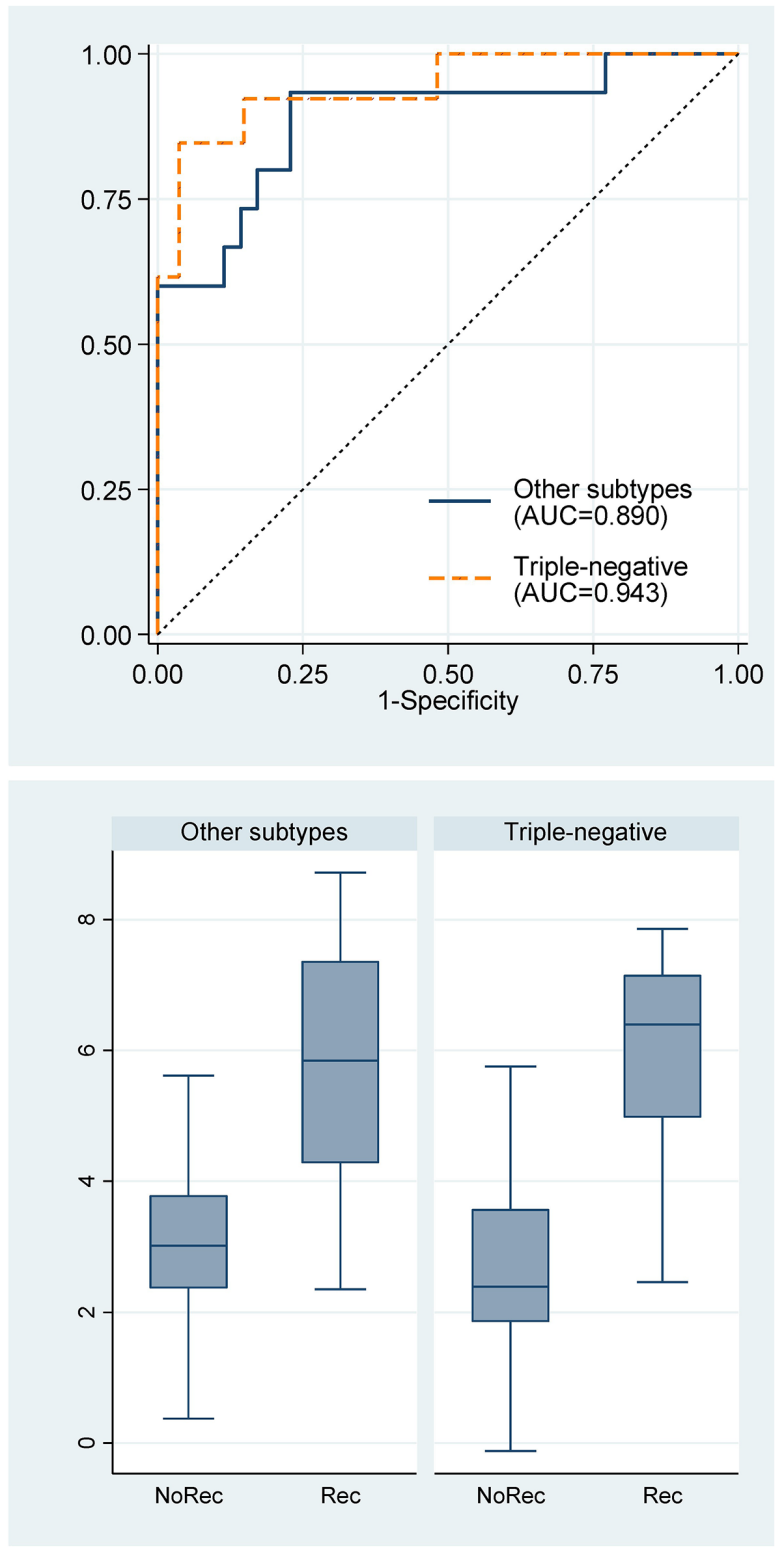
Receiver operating characteristic curves for the 7-miRNA signature and box plots of the 7-miRNA signature by breast cancer subtypes. Rec, recurrence; NoRec, no recurrence; TN, triple-negative subtype; Non-TN, other subtypes

Furthermore, stratified analysis showed that the miRNA signature was applicable to both triple-negative breast cancer (n=40) and other subtypes of breast cancers (ER+/PR+/Her2-, n=39; ER+/PR+/Her2+, n=5; ER-/PR-/Her2+, n=6) (Figure 6). The concordant indexes for triple-negative breast cancer and other subtypes were not statistically significant different.

### Compared with circulating microRNAs from non-cancer women

**Figure 7:**
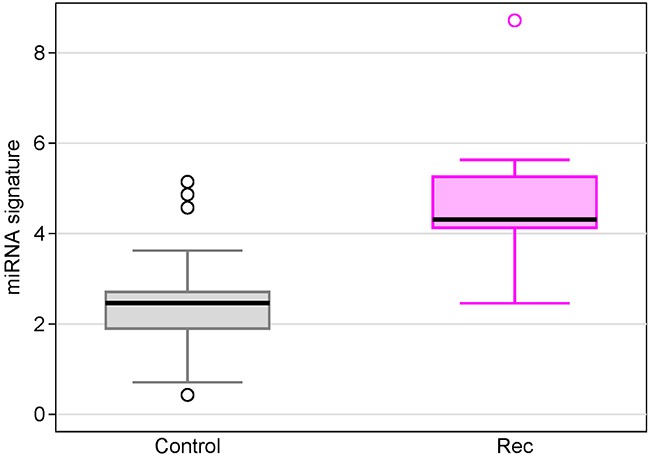
Box plots of the 7-miRNA signature in breast cancer patients with recurrence compared with non-cancer controls

In order to understand the baseline status of the miRNA expression in healthy women, we measured miRNA expressions in sera from 31 non-cancer controls using Exiqon's miRCURY microRNA Ready-to-Use PCR Human panels I+II. We compared serum 7 miRNAs levels individually as well as the miRNA signature between recurrent breast cancer patients and non-cancer controls. We found the miRNA signature in recurrent patients was 5.14-fold higher than non-cancer controls (Figure 7, p=1.1×10^−5^). At the cutoff point of 4.2 for the miRNA signature, 28 women were correctly classified as normal (specificity = 90.3%). The individual miRNA levels between the two groups were either statistically significant or trend towards significant (Supplementary Table 1).

### Reproducibility of individual microRNAs and microRNA signature

**Figure 8:**
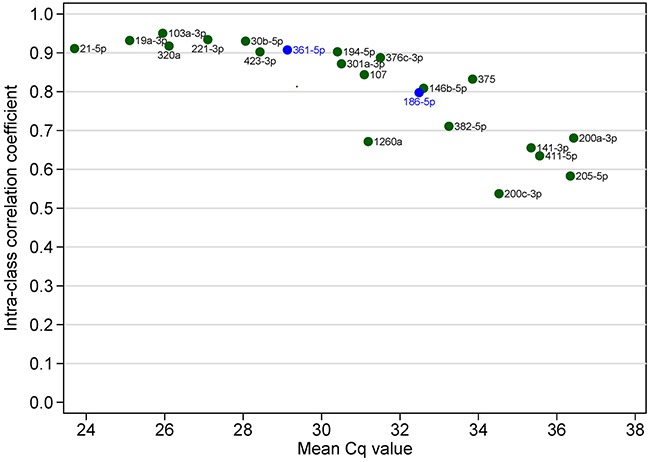
Intra-class correlation coefficient (as the index of reproducibility) of 22 individual miRNAs in the validation phase

**Figure 9:**
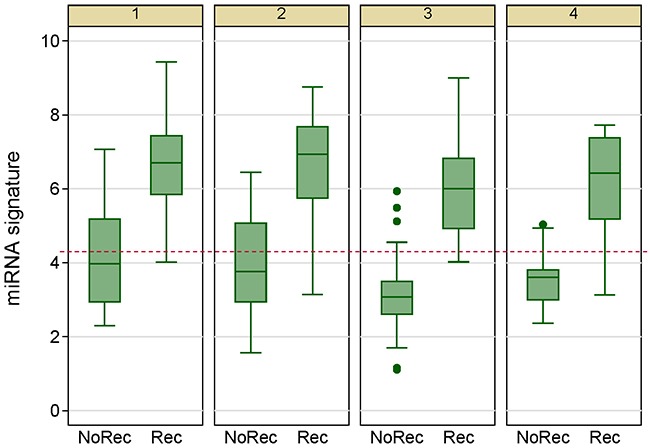
Box plots of the 7-miRNA signature in 4 repeated experiments show that the signature can reproducibly distinguishes patients with and without breast cancer recurrence

In the validation phase, expression of miRNAs was measured in quadruplicates so we can examine measurement reliability of the 20 candidate miRNAs and two endogenous control miRNAs. All miRNAs had ICC above 0.5 and nine miRNAs had ICC above 0.9. As expected, ICC was negatively correlated with mean Cq value, i.e. abundant miRNAs were more reliably measured than less abundant miRNAs in serum (Figure 8). We also evaluated the reliability of the 7-miRNA signature and found that it can reproducibly distinguish recurrent from non-recurrent patients (Figure 9). The ICC for the 7-miRNA signatures was 0.780, which means that the reliability coefficient for the 7-miRNA signature would be 0.934 if the qRT-PCR experiments were done in quadruplicates and 0.914 if the qRT-PCR experiments were done in triplicates.

### Pathway Analysis of Significant MicroRNAs

In the KEGG pathway analysis of the union of targeted genes of the 32 miRNAs identified in the discovery phase, we found that 72 pathways were enriched, with the top pathway being the “microRNAs in cancer” pathway (FDR = 4.0×10^−68^). The overall test for pathways of cancer was also significant (FDR = 1.1 × 10^−6^). Note that “breast cancer pathway” was not exist in KEGG database and two miRNAs had no experimentally validated gene targets, but we found several pathways related to breast cancer, such as “estrogen signaling pathway” and “ErbB signaling pathway” (Supplementary Table 2). In the pathway analysis of the intersection of targeted genes of at least 8 miRNAs (out of 32 miRNAs), we found 18 pathways were enriched, with the top pathway being “pathways in cancer” (Supplementary Table 3).

## DISCUSSION

In this study, we have identified seven miRNAs (miR-194-5p, miR-205-5p, miR-21-5p, miR-375, miR-376c-3p, miR-382-5p, and miR-411-5p) in serum that can distinguish patients with recurrence from those without among breast cancer patients. We have developed a 7-miRNA signature, which provided an excellent discriminating ability with a concordance index of 0.914. These microRNAs can be quantified reliably using a qRT-PCR method with less than 0.2 ml of serum.

Of the seven miRNAs identified in this study, two miRNAs (miR-21-5p, miR-375) in circulation have been found to be associated with prognosis of breast cancer. Candidate miRNA studies found that elevated miR-21-5p expression in serum was correlated with poor prognosis in breast cancer [11, 18], which is consistent with our finding that serum miR-21-5p was related to recurrence. Furthermore, miR-21-5p in breast tumors was also associated with poor survival in breast cancer [19], and circulating miR-21-5p predicted poor survival in other cancers [20]. As miR-21-5p overexpression increased cell growth, invasion and migration, and reduced apoptosis [21, 22], through downregulation of several tumor suppressor genes such as *PTEN*, *TPM1*, and *PDCD4* [23–25], miR-21-5p is likely to be a true prognostic factor for breast cancer and other cancers. Madhavan et al found that plasma level of miR-375 was higher in circulating tumor cells (CTC)-positive metastatic breast cancer patients than healthy controls [16]. Wu et al found lower serum level of miR-375 was associated with recurrence among locally advanced breast cancer patients in the discovery cohort but could not confirm this finding in their validation cohort [26]. In line with Madhavan et al but different from Wu et al, we found that serum miR-375 was positively associated with recurrences. A recent study showed that miR-375 was involved epithelial-to-mesenchymal transition in breast cancer cell lines, and thus related to metastasis [27].

To the best of our knowledge, the other five circulating miRNAs have not been reported to be associated with the prognosis of breast cancer, but they have been indicated as possible early detection markers or implicated in carcinogenesis process. Although miR-205-5p was considered a tumor suppressor [28], its role in breast cancer development and progression is unclear; one study found serum miR-205-5p was lower in breast cancer patients than healthy controls [29], but another study showed an opposite relationship [30]. One study found miR-376c-3p was elevated in serum of breast cancer patients compared to healthy controls [31], while another study showed that miR-382-5p in serum was higher in breast cancer patients than healthy controls [32]. Another study showed that miR-411-5p was lower in serum of breast cancer patients than that of healthy controls [14]. Lastly, circulating miR-194-5p was associated with colorectal cancer diagnosis [33], prostate cancer progression [34], and esophageal cancer [35], although the direction of association varied by cancer sites. Taken together, all seven miRNAs identified in our study are biologically plausible biomarkers.

In this study, we demonstrated that the 7-miRNA signature has better performance in predicting breast cancer recurrence than individual miRNAs. Recently, Sahlberg et al reported a 4-miRNA signature (miR-18b, miR-103, miR-107, and miR-652) that predicted relapse and overall survival for triple-negative breast cancers, with a concordance index of 0.810 [17]. Similar to our study, the study used serum samples of 60 breast cancers with Exiqon's RT-PCR array. In the discovery phase of our study, high expression of miR-103 and miR-107 were associated with recurrence, which is consistent with Sahlberg et al, though the miRNAs were no longer significant in the validation phase of our study. One possible reason for lack of overlap in miRNA signatures between the two studies is that breast cancer is a heterogeneous disease. Our study included both estrogen receptor (ER) positive and negative cancer. Although we found the 7-miRNA signature had prognostic capacity in both triple-negative breast cancers and other subtypes of breast cancers (mainly ER+/HER2-), the study was underpowered for comparing different subtypes. Another possible reason is that neither study is large enough to find all important prognostic miRNAs in circulation. The origin of tumor-associated miRNAs in circulation is not very clear [36]. They may be secreted by tumor cells in the primary site, circulating tumor cells, or metastatic lesions; they may also originate from immunocytes in the tumor microenvironment. Different sources of circulating miRNAs may reflect every aspect of tumor progression [36–38]. In the KEGG pathway analysis, we found that the 29 miRNAs we identified in the discovery phase were highly enriched to regulate genes in the cancer pathways, suggesting that they are biologically plausible candidates. Therefore, larger confirmative studies and meta-analysis of published data on circulating miRNAs hold a promise to generate better, reproducible prognostic signature for breast cancer.

This study has several strengths, including systematic miRNome discovery and validation approach, sensitive qRT-PCR assays, stringent quality controls in sample collection and processing, and blinded manner in experiments. The concentration of miRNAs in serum and plasma are highly concordant among different individuals [9, 10, 39], but proper operating procedures for blood collection should be followed to avoid hemolysis and disturbance of platelets. We used gel-separation method for serum collection, which can minimize cellular contamination.

Several limitations should be considered in interpreting our study findings. First, it is challenging to quantify miRNAs in serum because of the low abundance of miRNA in circulation and this may be an important reason why previous high-throughput miRNA profiling studies of circulating miRNA are inconsistent [40]. The reliability of measurement is less optimal for miRNAs less representative in serum (e.g. mean Cq>34). For example, we found that 3 members of the miR-200 family (miR-200a, miR-200c, and miR-141), all less abundant in serum, were significant in the discovery phase but were not statistically significant in the validation phase (the directions of association remained the same). These 3 miRNAs have been found to be associated with CTC-positive metastatic breast cancer [16]. So we may have false negative results because of measurement error. One simple solution is to increase the volume of serum/plasma, e.g. increasing from 0.2 ml to 2 ml. Second, there is no consensus in terms of normalization strategies for cell-free RNAs in circulation [41]. Unlike cellular RNAs from tumors, housekeeping genes such as small nucleolar RNA U6 may not be consistently detectable in serum. For instance, snRNA U6 was not detectable in half the samples in our study. In the discovery phase, we used global means to do the normalization as several hundred miRNAs were profiled. In the validation phase, we chose two miRNAs (miR-361-5p and miR-186-5p) as endogenous controls using stringent criteria. We are sure that the two endogenous control miRNAs have no association with breast cancer recurrence and their expression in the discovery and validation phase are quite similar. However, the two endogenous control miRNAs might be breast cancer specific, rather than universally applicable to other circulating miRNA studies. Third, we only assessed the reproducibility of qRT-PCR experiment but there may be variation due to RNA extraction. Further studies that have separately RNA extraction in different days are desirable to evaluate reproducibility of circulating miRNAs. Lastly, the study included diverse samples as we considered this study still in the early phase of biomarker development. We have carefully matched patients with and with recurrence according to age and subtype, and we adjusted for unmatched clinical factors in multivariable analysis, so the results are less prone to bias. However, our statistical power for detecting subtype-specific biomarker is limited.

There are several models of cancer metastatic process, including (a) the traditional model that the metastatic capacity is a late, acquired event in tumorigenesis, (b) the model that the ability to metastasize is an early, inherent property of the breast tumors, (c) the model that metastasis is a mechanical, random process, and (d) the model that tumor DNA in circulating plasma transfects to susceptible cells in distant organs [42]. Each model had its supporting evidences from experimental or observational studies, suggesting that the cancer metastatic cascade is a complex process [42]. The clinical implication of these distinct models is related to when we can predict cancer metastasis: at diagnosis or later. An accurate prediction of prognosis at diagnosis is critical for clinicians to tailor the treatment plan to maximize efficacy and reduce unnecessary toxicities from treatments, while early detection of metastasis after initial treatment provides an important window of opportunity because new targeted therapies may be more effective in treating early recurrent cancer before the cells have had the chance to acquire additional mutations leading to resistance. In this study, we included serum samples at diagnosis and at time around metastatic recurrences, and we found that circulating miRNAs at both time points were associated with recurrences, providing some supporting evidence for the theory that metastasis is an early event. Biomarkers such as miRNAs in tumor samples could provide complementary information to circulating miRNA. On the other hand, the prediction at baseline is not perfect so it is necessary to continue monitoring cancer progression after treatment.

In conclusion, our pilot study findings suggest that microRNAs in circulation can provide a less-invasive, inexpensive “liquid-biopsy” method to monitor breast cancer metastasis. We envision that our miRNA signature for recurrence is promising in clinical application as we have demonstrated its excellent discriminating capacity, good reproducibility, and difference from healthy controls. However, further prospective, longitudinal studies are desirable to evaluate the clinical potential of circulating miRNAs as continuous cancer recurrence surveillance. Another direction of further research is to assess the relationship between circulating miRNAs and other biomarkers such as circulating tumor cells and circulating tumor DNAs.

## MATERIALS AND METHODS

### Sample collection and processing

The study was approved by the Institutional Review Board of the University of Chicago. Breast cancer patients were selected randomly from the consecutive series of nearly 2700 patients enrolled in the Chicago Multiethnic Breast Cancer Epidemiologic Cohort at the University of Chicago. We used a case-control study nested within the cohort. Cases were histologically confirmed invasive breast cancer patients who developed locoregional or distant recurrences and there are two groups of cases according to the time of serum collection; One group of cases had sera collected after cancer diagnosis and before surgery (labeled as “Rec-B” group), whereas the other group of cases had sera collected around the time of recurrence (labeled as “Rec-A” group). Controls were invasive breast cancer patients who had no recurrence during a median follow-up of 36 months (labeled as “NoRec” group) and were matched to cases with respect to age and proportion of triple-negative cancer subtype. For patients in the control group, sera were collected after diagnosis and before surgery. We purposely included two groups of recurrent cases and both triple-negative and hormone receptor positive subtypes, in order to have a wide spectrum of tumors, as suggested in the guideline for the early phase of biomarker development [43]. Demographic and clinopathological characteristics were collected in these patients. Histological grade was determined by modified Bloom-Richardson grading system [44].

In order to understand the “baseline” status of miRNA expression, we also included 31 non-cancer controls who did not have a breast cancer. These non-cancer controls were also recruited at the University of Chicago hospitals for mammographic screening or breast lumps which were diagnosed as benign breast diseases.

After informed consent, whole blood was collected in red/gray SST Serum Separator Tubes (BD Vacutainer). Collected blood were allowed to clot at room temperature for 30 minutes, and then centrifuged at 4°C at 2500 rpm for 10 minutes. Serum layers were collected, separated into 3 aliquot tubes, and immediately frozen at −80°C until use. Total RNA were extracted from 200 ul serum using miRNeasy Serum/Plasma kit (QIAGEN) following the manufacturer's protocol. We used 1 μg of MS2 bacteriophage rRNA (Roche) as the carrier RNA to increase yield. Three 22nt synthetic RNAs (UniSp2, UniSp4, and UniSp5) from Exiqon were added to each reaction after lysis and before phase separation. RNA quality was evaluated using the miRCURY microRNA QC PCR Panel (Exiqon) and samples that did not meet the quality control measures were excluded. In particular, we excluded hemolysed samples as indicated by high ratio of hsa-miR-451a to hsa-miR-23a (ΔCq>7), because circulating, cell-free miRNAs mainly come from blood cells in hemolysis samples [45].

### MicroRNA Quantification by Quantitative RT-PCR

The study was conducted in two phases. In the discovery phase, expression of miRNAs from sera was evaluated using miRCURY LNA Universal RT microRNA Ready-to-Use PCR Human panels I+II V3.M (Exiqon), which contains assays for 752 human microRNAs. Reverse transcription (RT) was performed using the Universal cDNA synthesis kit II (Exiqon) with the addition of two spike-ins (UniSp6 and cel-miR-39-3p) to the RT reaction. For quantitative PCR (polymerase chain reaction), 1:80 water diluted cDNA products were mixed at a 1:1 ratio with the ExiLENT SYBR Green Mastermix (Exiqon) that had Rox Reference Dye (Life Technologies) previously added to it. For quality control purpose, one RNA sample was measured twice and a sample containing nuclease-free water and carrier RNA was profiled as negative control. GenEx software (Multi-D) was used for data pre-processing including inter-plate calibration, evaluation of isolation and reverse transcription efficiency, setting specific cut-offs for negative control microRNA Cq values, and duplicates averaging. We performed global mean normalization with the assumption that the majority of miRNAs were not related to disease status so can reflect overall quantity of RNA added. MicroRNAs with a Cq value > 37 were deemed to be not detected.

In the validation phase, miRNAs that were differentially expressed between patients with and without recurrences in the discovery phase were further validated in independent serum samples using individual microRNA LNA PCR primer sets (Exiqon). In brief, RNA samples were reverse transcribed in duplicates. Then all cDNA products were prepared in duplicate PCR reactions following manufacturer's instructions. It is not appropriate to perform global mean normalization in validation phase because only recurrence-differentiated miRNA were chosen. Instead, we chose miR-361-5p and miR-186-5p as endogenous control miRNAs for normalization because the two miRNAs fulfilled the following criteria: a) high expression in serum, b) expressed stably across samples evaluated by Normfinder and geNORM [46, 47], c) not differentially expressed between study groups in the discovery phase, d) strongly correlated with the global mean in the discovery phase, and e) not related to breast cancer based on literatures of population studies.

### Statistical analysis

In the discovery phase, we first excluded miRNAs that were detectable in less than half of the samples as these miRNAs are usually unreliably measured. Then we normalized Cq values to global mean. Here, high Cq value indicates low expression. When a miRNA was undetected in a sample, its Cq value was set to the maximum Cq across all samples plus 1 (usually set to 38). Moderated t test was used to identify miRNAs differentially expressed between patients with recurrence and those without recurrence. The variances in calculating of the t statistics were moderated using empirical Bayes approach [48]. Benjamini-Hochberg's false discovery rate method was used to correct for multiple testing. All miRNAs with p<0.05 were candidate miRNAs and we chose independent miRNAs among these candidate miRNAs for further validation. Specifically, we only chose the miRNA with low mean Cq value (i.e. the more reliable one in PCR experiment) if two were highly correlated with correlation coefficient>0.7. Hierarchical clustering analysis with Spearman correlation as the similarity measure was conducted to summarize the overall pattern of miRNA expression.

In the validation phase, we first normalized Cq values of each miRNA to endogenous control miRNAs. Then we used linear models for microarray data followed by moderated t test to validate which miRNAs were differentially expressed among the three study groups: samples obtained at diagnosis for patients without recurrence (the “NoRec” group), samples obtained at diagnosis for patients with recurrence (the “Rec-B” group), and samples obtained at recurrence for patients with recurrence (the “Rec-A” group). As preliminary analysis showed that the significant miRNAs were similar between the last two groups, we conducted further analysis combining the two recurrent groups. As the significant miRNAs identified in the univariate analysis may be correlated and high-dimensionality may cause overfitting, we used an elastic net penalized logistic regression to create a miRNA signature for recurrence [49]. Cross-validations were used to tune the penalty parameters. The miRNA signature score for subject *i* was calculated as follows: $M\;SS_{i}=\sum_{K=1}^{K}W_{k}S_{\text{ik}}$, where *W_k_* is the multivariable-adjusted log odds ratio for miRNA *k* from penalized regression and *S_ik_* is the normalized miRNA expression. Receiver operating characteristic (ROC) curves were built for each miRNA and the miRNA signature, and area under the ROC curve (AUC), i.e. concordance index, was calculated to indicate the discriminating capacity. Notably, we re-nomalized miRNA expression in the discovery phase using the two selected endogenous control miRNAs before pooling data of the two phases in order to calculate pooled ROC curve. We also examined the reproducibility of individual miRNAs and the miRNA signature by calculating intra-class correlation coefficient (ICC) using random effect models. Using the Spearman-Brown formula [50, 51], we calculated the reliability coefficient from ICC. Statistical analysis was carried out using STATA v13 (Statacorp) and Bioconductor packages including NormqPCR, HTqPCR, Limma, and Penalized, based on open environment R 3.1.1 (www.r-project.org, www.bioconductor.org).

### Pathway analysis

In order to understand the biological significance of miRNAs that were significantly associated with breast cancer recurrence, we conducted KEGG pathway analysis using DIANA-miRPath v3.0 (www.microrna.gr/miRPathv3) [52]. We used experimentally validated targeted genes of the miRNAs from TarBase v7.0 [53] to examine the enrichment of biological pathways. We calculated the union of targeted genes by at least one selected miRNAs, and the intersection of targeted genes by at least a quarter of all selected miRNAs.

## SUPPLEMENTARY FIGURE AND TABLES


