# Supplementary material for: Identification of a circulating MicroRNA signature to distinguish recurrence in breast cancer patients
Source: Oncotarget. 2016 Jul 8;7(34):55231–48. doi: 10.18632/oncotarget.10485 (PMC5342414; doi:10.18632/oncotarget.10485)
Supplement: Supplementary file 1 [file oncotarget-07-55231-s001.pdf]

SUPPLEMENTARY FIGURE AND TABLES

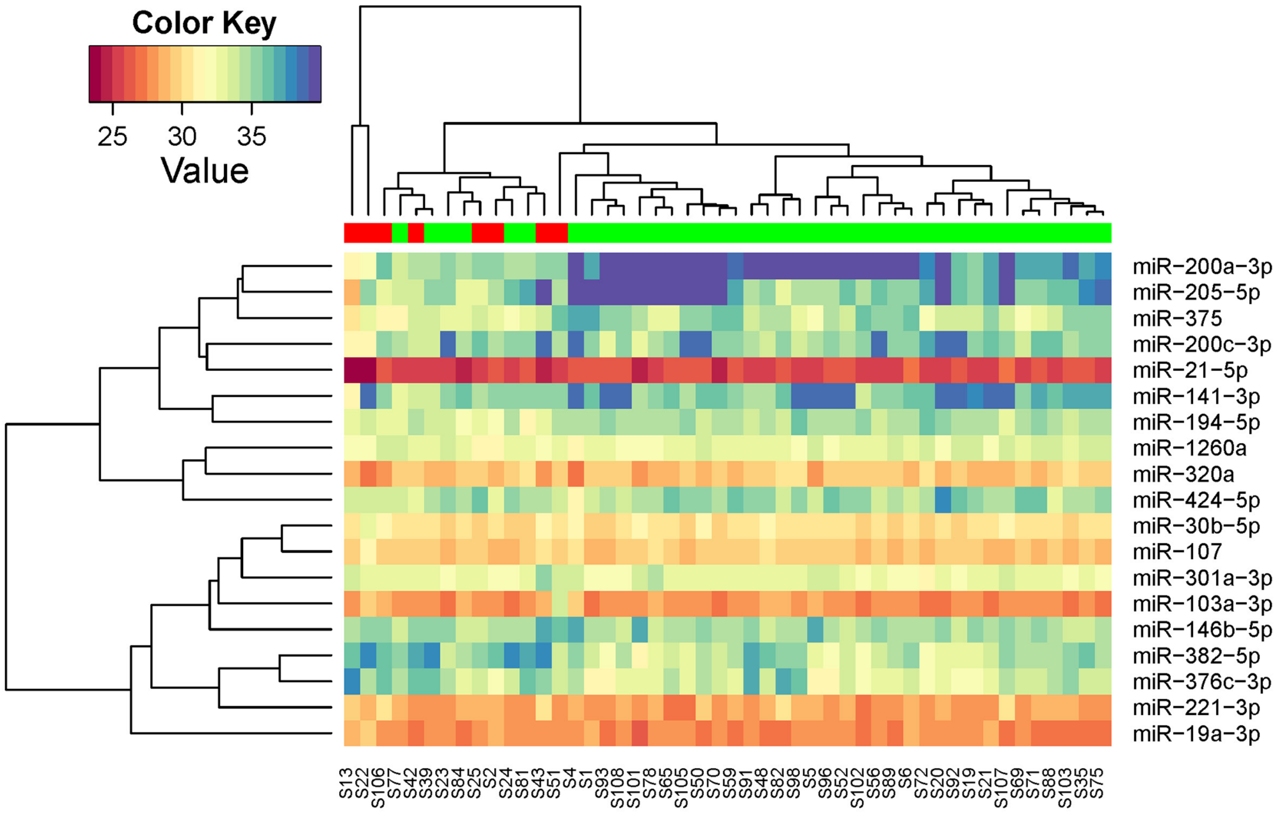

Supplementary Figure 1: Unsupervised hierarchical clustering with Pearson distance metric, based on the 19 miRNAs that were selected for further validation experiment (red: samples at recurrence from patients with recurrence; green: samples at diagnosis from patients without recurrence).

Supplementary Table 1: Normalized expression of 7 miRNAs in sera of recurrent breast cancer patients and non-cancer controls

	Non-cancer controls (N=31)		Recurrent patients (n=8)		Fold Difference	P value
	Mean	SD	Mean	SD		
miR-194-5p	-3.66	0.83	-3.10	0.94	1.48	0.11
miR-205-5p	-6.81	2.17	-3.74	3.16	8.42	0.001
miR-21-5p	4.47	0.48	5.27	1.11	1.75	0.001
miR-375	-3.56	1.48	-2.75	1.70	1.76	0.18
miR-376c-3p	-3.20	1.07	-4.61	1.83	0.38	0.11
miR-382-5p	-3.75	1.23	-5.89	1.44	0.23	3.0E-04
miR-411-5p	-7.14	1.98	-8.47	1.48	0.40	0.08

Supplementary Table 2: Significantly enriched KEGG pathways for the 32 microRNAs identified in the discovery phase

KEGG pathway	FDR	# of genes	# of miRNAs
**MicroRNAs in cancer**	**4.02E-68**	**143**	**29**
Proteoglycans in cancer	1.36E-21	162	30
Cell cycle	9.74E-12	105	29
Viral carcinogenesis	1.75E-11	156	30
Endocytosis	5.13E-11	161	29
Hepatitis B	5.13E-11	111	30
Pancreatic cancer	6.32E-10	61	30
Renal cell carcinoma	1.94E-08	59	29
Fatty acid elongation	4.30E-08	18	18
Adherens junction	4.30E-08	63	28
Spliceosome	4.65E-08	104	29
Chronic myeloid leukemia	1.72E-07	64	30
Glioma	2.48E-07	53	30
FoxO signaling pathway	2.72E-07	109	30
**p53 signaling pathway**	**3.63E-07**	**61**	**29**
Transcriptional misregulation in cancer	3.63E-07	128	29
Prostate cancer	4.70E-07	77	30
Non-small cell lung cancer	5.22E-07	49	29
Protein processing in endoplasmic reticulum	5.22E-07	131	30
Hippo signaling pathway	5.22E-07	108	30
Colorectal cancer	6.20E-07	54	29
Other types of O-glycan biosynthesis	1.11E-06	24	21
**Pathways in cancer**	**1.11E-06**	**272**	**30**
N-Glycan biosynthesis	3.72E-06	40	25
Ubiquitin mediated proteolysis	5.33E-06	105	29
Endometrial cancer	6.36E-06	46	30
Bladder cancer	4.47E-05	35	28
**ErbB signaling pathway**	**4.47E-05**	**67**	**30**
HTLV-I infection	9.88E-05	182	29
Acute myeloid leukemia	0.000105	48	27
Bacterial invasion of epithelial cells	0.000105	60	28
Lysine degradation	0.000106	37	26
Neurotrophin signaling pathway	0.000106	91	30
Fatty acid metabolism	0.00014	30	23
Central carbon metabolism in cancer	0.000251	53	29
Prion diseases	0.000268	22	25

KEGG pathway	FDR	# of genes	# of miRNAs
Thyroid hormone signaling pathway	0.000268	86	30
Thyroid cancer	0.000297	25	24
TGF-beta signaling pathway	0.000304	58	30
Insulin signaling pathway	0.000616	103	30
RNA degradation	0.000708	61	28
TNF signaling pathway	0.000708	82	29
HIF-1 signaling pathway	0.000812	79	30
MAPK signaling pathway	0.000812	173	29
**mTOR signaling pathway**	**0.000812**	**51**	**29**
Small cell lung cancer	0.000878	66	28
Non-alcoholic fatty liver disease (NAFLD)	0.001353	108	28
Signaling pathways regulating pluripotency of stem cells	0.001665	98	30
Oocyte meiosis	0.001711	78	27
RNA transport	0.002663	117	29
Focal adhesion	0.002846	144	30
Sphingolipid signaling pathway	0.002963	87	30
**Estrogen signaling pathway**	**0.003272**	**69**	**29**
Apoptosis	0.003872	64	30
Regulation of actin cytoskeleton	0.003872	140	30
Glycosaminoglycan biosynthesis - keratan sulfate	0.005643	12	17
Melanoma	0.005643	52	30
Shigellosis	0.007605	47	27
Axon guidance	0.010275	87	28
**PI3K-Akt signaling pathway**	**0.011426**	**210**	**30**
Epstein-Barr virus infection	0.013971	137	29
Huntington’s disease	0.023394	122	29
Progesterone-mediated oocyte maturation	0.027642	63	30
Fc gamma R-mediated phagocytosis	0.032015	64	30
Gap junction	0.037212	58	28
VEGF signaling pathway	0.037838	45	28
Ribosome	0.039186	92	28
NF-kappa B signaling pathway	0.042572	57	27
mRNA surveillance pathway	0.042572	65	29
Choline metabolism in cancer	0.042572	72	29
Wnt signaling pathway	0.043012	91	29
Chagas disease (American trypanosomiasis)	0.049234	71	30

KEGG, Kyoto Encyclopedia of Genes and Genomes; FDR, false discovery rate

Supplementary Table 3: Significantly enriched KEGG pathways for the intersection of targeted genes of at least 8 microRNAs

KEGG pathway	FDR	# of genes	# of miRNAs
**Pathways in cancer**	**7.41E-05**	**28**	**29**
Lysine degradation	0.000125	7	24
Colorectal cancer	0.000228	9	26
p53 signaling pathway	0.000228	14	27
**MicroRNAs in cancer**	**0.001093**	**30**	**29**
Hepatitis B	0.002413	16	28
Glioma	0.014811	9	28
Thyroid hormone signaling pathway	0.014811	14	29
Proteoglycans in cancer	0.014811	18	29
Chronic myeloid leukemia	0.016312	10	26
Hippo signaling pathway	0.016312	17	27
PI3K-Akt signaling pathway	0.016312	27	29
Epstein-Barr virus infection	0.024709	22	29
Bladder cancer	0.026803	8	26
Focal adhesion	0.03458	20	29
Prostate cancer	0.034792	12	29
FoxO signaling pathway	0.034865	14	29
Cell cycle	0.037645	16	28

The 28 genes in the cancer pathways jointly targeted by at least 8 miRNAs:

ABL1, BCL2, CASP3, CCND1, CDK6, CDKN1A, CRKL, E2F1, E2F3, MDM2, MYC, PTEN, VEGFA, CTNNB1, FGF2, FN1, GSK3B, HIF1A, HSP90AA1, HSP90AB1, IGF1R, ITGA6, JUN, LAMC1, MAPK8, NCOA4, STK4, TGFBR2

The 30 genes in microRNAs in cancer pathway jointly targeted by at least 8 miRNAs

ABL1, BCL2, CASP3, CCND1, CDK6, CDKN1A, CRKL, E2F1, E2F3, MDM2, MYC, PTEN, VEGFA, BCL2L11, BMPR2, CCND2, CCNG1, CDC25A, DDIT4, DICER1, FOXP1, GLS, HMGA2, IGF2BP1, MARCKS, MCL1, NOTCH2, SOX4, THBS1, TRIM71

KEGG, Kyoto Encyclopedia of Genes and Genomes; FDR, false discovery rate
